# Generation of Otic Lineages from Integration-Free Human-Induced Pluripotent Stem Cells Reprogrammed by mRNAs

**DOI:** 10.1155/2020/3692937

**Published:** 2020-03-01

**Authors:** Sarah L. Boddy, Ricardo Romero-Guevara, Ae-Ri Ji, Christian Unger, Laura Corns, Walter Marcotti, Marcelo N. Rivolta

**Affiliations:** ^1^Centre for Stem Cell Biology, University of Sheffield, Sheffield S10 2TN, UK; ^2^Department of Biomedical Sciences, University of Sheffield, Sheffield S10 2TN, UK

## Abstract

Damage to the sensory hair cells and the spiral ganglion neurons of the cochlea leads to deafness. Induced pluripotent stem cells (iPSCs) are a promising tool to regenerate the cells in the inner ear that have been affected by pathology or have been lost. To facilitate the clinical application of iPSCs, the reprogramming process should minimize the risk of introducing undesired genetic alterations while conferring the cells the capacity to differentiate into the desired cell type. Currently, reprogramming induced by synthetic mRNAs is considered to be one of the safest ways of inducing pluripotency, as the transgenes are transiently delivered into the cells without integrating into the genome. In this study, we explore the ability of integration-free human-induced pluripotent cell lines that were reprogrammed by mRNAs, to differentiate into otic progenitors and, subsequently, into hair cell and neuronal lineages. hiPSC lines were induced to differentiate by culturing them in the presence of fibroblast growth factors 3 and 10 (FGF3 and FGF10). Progenitors were identified by quantitative microscopy, based on the coexpression of otic markers PAX8, PAX2, FOXG1, and SOX2. Otic epithelial progenitors (OEPs) and otic neuroprogenitors (ONPs) were purified and allowed to differentiate further into hair cell-like cells and neurons. Lineages were characterised by immunocytochemistry and electrophysiology. Neuronal cells showed inward Na^+^ (*I*_Na_) currents and outward (*I*_k_) and inward K^+^ (*I*_K1_) currents while hair cell-like cells had inward *I*_K1_ and outward delayed rectifier K^+^ currents, characteristic of developing hair cells. We conclude that human-induced pluripotent cell lines that have been reprogrammed using nonintegrating mRNAs are capable to differentiate into otic cell types.

## 1. Introduction

Hearing loss has a huge impact on quality of life, as well as an economic cost for society. Advances in hearing aid and cochlea implant technologies offer partial functional recovery for some, but for many, there is no curative treatment. Generation of biological therapeutic agents to replace damaged cellular components of the inner ear remains a goal of regenerative medicine, which could eventually lead to treatment options for excluded patients and better outcomes for those currently dependent on electronic devices. Key work undertaken on mouse and human stem cells has made significant advances towards this objective [1–6]. Furthermore, progenitors derived from human embryonic stem cells (hESCs) have been transplanted into the cochleae of deafened gerbils, eliciting functional recovery [6].

The identification of potential sources of “adult stem cells” within the body (reviewed in [7]) has raised hopes for autologous, patient-specific cellular therapies. While this concept is attractive both from ethical and patient compatibility perspectives, to date, adult stem cell populations have proven difficult to manipulate to raise progenies beyond their natural potential. These cell types generally show far less differentiation plasticity than their embryonic counterparts, limiting their widespread application. For example, studies attempting to steer mesenchymal stem cells towards an otic fate have provided preliminary proof-of-principle, but differentiation is limited and yields of otic progenitors are very low using current protocols [8–10].

Adult stem cells have been overshadowed in recent years by the advances in reprogramming technology and the development of induced pluripotent stem cells (iPSCs). The ability to reprogram a somatic cell into a pluripotent state was initially described in 2006 by Takahashi and Yamanaka, through the exogenous expression of four genes, Oct3/4, Sox2, c-Myc, and Klf4 [11], spearheading a revolution in the field. Since its inception, this technology has progressed quickly, leading to increased reprogramming efficiency, applicability to an ever-expanding repertoire of cell types, and circumvention of genome integration of exogenous genes (comprehensively reviewed by [12, 13]).

Before iPSCs can be considered viable agents for clinical application, concerns about the genetic integrity of the induced cells need addressing. Viral vectors used during conventional reprogramming can cause insertional mutagenesis and the activation of oncogenes. In order to avoid this problem, virus-free and integration-free methods have been developed, such as the use of episomal plasmids [14, 15] or mRNA-based reprogramming [16]. Messenger RNA reprogramming would appear to be the safer of the two, since it has been recently reported that a clinical-grade iPSC line derived with episomal plasmid vectors presented altered gene copy numbers, preventing its administration to the patient [17].

Otic differentiation from human pluripotent stem cells has been obtained using embryonic stem cells [3, 6, 18, 19], and more recently, a few reports have employed iPSCs [20–25]. However, these early reports on iPSCs have mostly used integrative retroviral or lentiviral vectors. While it is expected that the mRNA transfection method of cellular reprogramming would generate iPSCs with equivalent differentiation potential to those generated via viral transduction, it has not yet been explored if cells reprogrammed with mRNAs would have a similar ability to differentiate into otic lineages.

## 2. Materials and Methods

### 2.1. Cell Derivation and Maintenance

The four iPSC lines used in this study were derived from human foreskin fibroblasts (CRL-2429, ATCC) by two different methods. FF1 and FF5 lines were transduced with integrating lentiviral vectors encoding *SOX2*, *OCT4*, *LIN28*, and *NANOG*, while MIFF1 and MIFF3 lines were reprogrammed using nonintegrating mRNAs encoding *SOX2*, *OCT4*, *LIN28*, *KLF4*, and *c-MYC* (according to the manufacturer's instructions, Stemgent, USA). All four lines have typical ES cell morphology and are confirmed to express pluripotency markers such as SSEA4, Tra1-81, Oct4, and Nanog. All four lines had a normal 46, XY karyotype. Generation and characterisation of the lines reprogrammed with nonintegrating mRNAs were reported in [26], while characterisation of the lentivirus-reprogrammed lines is presented in the supplementary information (Supplementary [Supplementary-material supplementary-material-1]). All iPSC stocks were maintained on mouse-inactivated fibroblast feeders in KOSR medium (as described in [26]) at 37°C and 5% CO_2_.

### 2.2. iPSC Differentiation into Otic Progenitors

Differentiation protocols were as reported for hESCs on [6]. In brief, generation of otic progenitors was achieved by plating iPSCs in Dulbecco's Modified Eagle Medium: Ham's F12 (DMEM/F12) supplemented with 1x N2 and B27 (abbreviated as DFNB) (all Life Technologies, UK), FGF3 and FGF10 (both 50 ng/ml) (both R&D Systems, UK) onto laminin-coated tissue culture plastic. If cells were to be further differentiated along hair cell or auditory neuronal lineages, cultures were manually purified by removing cells lacking relevant characteristic progenitor morphologies, enriching specifically for either otic epithelial progenitor (OEP) or otic neuronal progenitor (ONP) phenotypes. Quantification of each progenitor colony type was performed by counting colonies with the characteristic morphologies at day 4 of differentiation, before the cleaning of undesired cells and when the separation between colonies is as its best. At least 3 randomly selected 20 mm^2^ fields were sampled from each T25 flask, and ten flasks were counted per line. Statistical comparisons were done using 2-way ANOVA. Results were normalised to 1 cm^2^ and reported as mean colony number/cm^2^ ± SEM.

### 2.3. iPSC-Derived Progenitor Differentiation towards Hair Cell and Sensory Neuronal Fates

Manually enriched populations of OEPs were dissociated using mild trypsin EDTA solution (1 : 80) (Sigma-Aldrich, UK) and seeded onto gelatin-coated tissue culture plastic in DFNB medium supplemented with 1 × 10^−6^ M retinoic acid (Sigma-Aldrich, UK) and 20 ng/ml epidermal growth factor (EGF) (R&D Systems, UK). Medium was replaced every other day, and cells were, after 14 days, either fixed in 4% paraformaldehyde or tested for electrophysiological responses.

ONP cultures were dissociated using trypsin solution (1 : 10) (Sigma-Aldrich, UK) and seeded onto gelatin-coated tissue culture plastic in DFNB medium supplemented with 20 ng/ml basic fibroblast growth factor (bFGF) (PeproTech, UK) and 500 ng/ml sonic hedgehog (Shh-C24II) (R&D Systems, UK). Medium was replaced every other day, with 10 ng/ml neurotrophin-3 (NT3) and 10 ng/ml brain-derived neurotrophic factor (BDNF) (both PeproTech, UK) added from day 3 onwards and Shh-C24II removed from day 5.

### 2.4. Electrophysiology Recordings

Whole-cell patch clamp recordings in voltage clamp mode were made from cultured cells using an Optopatch amplifier (Cairn Research) at room temperature. Cells were viewed using an upright microscope (Leica DMLFS, Germany) and were continuously superfused with extracellular solution (mM) containing 135 NaCl, 5.8 KCl, 1.3 CaCl_2_, 0.9 MgCl_2_, 0.7 NaH_2_PO_4_, 5.6 D-glucose, 10 HEPES-free acid, and 2 sodium pyruvate. MEM amino acid solution (50x, without L-glutamine) and MEM vitamin solution (100x) were added from concentrates (Fisher Scientific), and the pH was adjusted to 7.5. Soda glass patch pipettes coated with surf wax were filled with solution (mM) containing 131 KCl, 3 MgCl_2_, 1 EGTA–KOH, 5 Na_2_ATP, 5 HEPES–KOH, and 10 Na_2_ phosphocreatine and adjusted to pH 7.3. Data were acquired using pClamp software and a Digidata 1440A analogue-to-digital converter (Molecular Devices). Data were filtered at 2.5 or 5 kHz, sampled at 5 or 50 kHz, and stored on a computer for offline analysis using Clampfit and Origin (OriginLab) software. Cells were held at -64 mV or -84 mV, and positive and negative voltage steps in 10 mV nominal increments were applied. Averages are presented as mean ± standard error of the mean (SEM).

### 2.5. Fluorescence Staining

Cells previously fixed for 15 min at room temperature in Phosphate-Buffered Saline (PBS) with 4% paraformaldehyde were blocked with 0.1% Triton-X, 5% donkey serum, and 1% bovine serum albumin in PBS. The following primary antibodies were used in this study: SOX2 (1 : 100, Millipore), FOXG1, PAX2, HATH1 (ATOH1) (all 1 : 100, Abcam UK), PAX8 (1 : 100, Santa Cruz), POU4F3 (BRN3C, 1 : 50, Abnova), POU4F1 (BRN3A, 1 : 100, Chemicon), and B-tubulin III (1 : 100, Sigma). Secondary antibodies used were anti-mouse, anti-goat, or anti-rabbit Alexa Fluor 488 and 568 (Molecular Probes, Life Technologies, UK), while nuclei were counterstained with 4′,6-diamidino-2-phenylindole (DAPI) (Sigma). Cells were imaged either on an EVOS FL Cell Imaging System or using the IN Cell Analyzer 2000 system platforms (GE Healthcare). Quantitative immunofluorescence was performed on the IN Cell Analyzer using the Developer Toolbox. Approximately 100-200 fields per antibody staining condition and per cell line were analyzed, capturing between 1,400 and 15,000 cells per condition, per line. Statistical comparison across the different antibody conditions and reprogramming methods was done using 2-way ANOVA. Results are reported as mean% ± SEM. The anti-SSEA3, SSEA4, TRA-1-60, and TRA-1-81 antibodies used for flow cytometry were from BioLegend.

### 2.6. Quantitative Real-Time PCR (qPCR)

Quantitative real-time PCR was performed on a QuantStudio 12K Flex Real-Time PCR system (Invitrogen) using TaqMan primers and universal master mix (Invitrogen). Data was analyzed using the *ΔΔ*Ct method, *RPLP0* as a reference gene and normalised against undifferentiated cells from the same starting population. Data is reported either as relative expression (2^-*ΔΔ*Ct^) or as fold changes against the original population.

## 3. Results

### 3.1. iPSCs Can Be Directed towards an Otic Progenitor State

**Figure 1:**
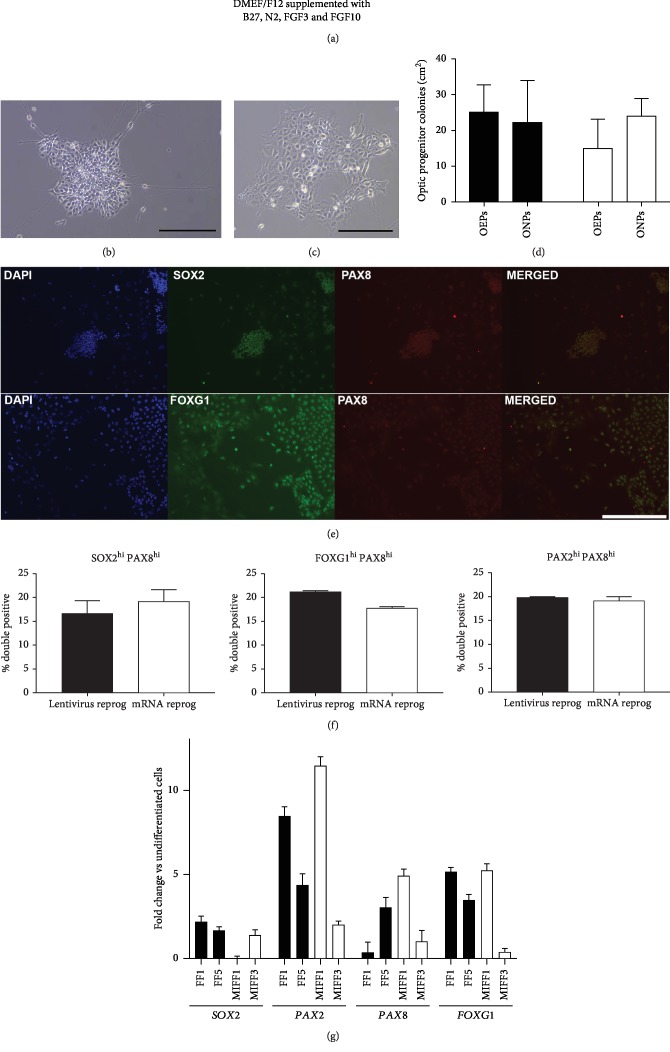
(a) Diagram showing the protocol for the generation of otic progenitors. Colonies of ONPs (b) and OEPs (c), displaying their typical morphology. Scale bar, 200 *μ*m. (d) Bar chart displaying the relative yields of OEPs and ONPs per reprogramming condition (dark bars are lentivirus-reprogrammed lines, while open bars are for mRNA-reprogrammed ones). (e) Top: ONP colony stained for SOX2 and PAX8. Bottom: otic epithelial progenitors showing coexpression of FOXG1 and PAX8. Images shown are from the FF1 cell line. Scale bar, 400 *μ*m. (f) Bar charts displaying percentages of double-positive otic progenitors, expressing high levels of SOX2/PAX8, FOXG1/PAX8, and PAX2/PAX8. (g) qPCR of otic progenitors showing relative levels of expression for *SOX2*, *PAX2*, *PAX8*, and *FOXG1.* Data shown as fold change against undifferentiated cells from the same starting population.

Otic induction has been demonstrated in hESCs using a two-step protocol, whereby an intermediate otic progenitor population is generated by exposure to FGF3 and FGF10 during cell seeding and subsequent monolayer culture, prior to directing them towards either a hair cell or neuronal fate (Figure 1(a)) [6]. The state of an otic progenitor is defined by the expression of key markers associated with the developing early otic placode (Figures 1(e) and 1(f)). Four iPSC lines, two generated by lentiviral transduction (FF1 and FF5) and two by mRNA reprogramming (MIFF1, MIFF3), were cultured for 12 days in FGF3 and FGF10 otic induction medium, before being probed with antibodies against PAX8, PAX2, SOX2, and FOXG1. In each case, cells were exposed to PAX8 antibodies in combination with those raised against one of the other markers (PAX2, SOX2, or FOXG1) to identify coexpression in individual cells (e.g., Figures 1(e) and 1(f)). Highly expressing cells were defined as those having fluorescence intensities above the 75^th^ percentile for each antibody independently (as described in [6]). Populations of cells that were highly positive for two markers (coexpression) were then identified as *bona fide* otic progenitors. Lentiviral-induced lines produced either 16.6% ± 2.8 SOX2hi/PAX8hi, 21.1% ± 0.23 FOXG1hi/PAX8hi, or 19.9% ± 0.09 PAX2hi/PAX8hi otic cells. On the other hand, mRNA-reprogrammed lines generated 19.2% ± 2.2 SOX2hi/PAX8hi, 17.7% ± 0.2 FOXG1hi/PAX8hi, or 19.3% ± 0.7 PAX2hi/PAX8hi cells. Overall, the yields of otic progenitors were not different between the two types of reprogramming technique used (*n* = 2, *p* > 0.05; Figure 1(f)). Moreover, a comparable yield of progenitors was observed when the independent cell lines were analyzed individually (Supplementary [Supplementary-material supplementary-material-1]).

The induction of otic marker gene expression was further validated by qPCR. *SOX2*, *PAX2*, *PAX8*, and *FOXG1* transcripts were upregulated in all lines when normalised against their starting undifferentiated population, displaying similar patterns of induction between the two reprogramming methods (Figure 1(g)).

The distinctive morphologies identified in differentiating hESC cultures and described as “otic neural progenitors” (ONPs) and “otic epithelial progenitors” (OEPs) were also observed in the iPSC cultures (Figures 1(b) and 1(c)). As the quantification described above did not discriminate between these two different progenitor types, we counted the different colonies obtained based on their morphologies. The yields of OEPs and ONPs were comparable between the lentiviral- and the mRNA-reprogrammed lines, with lentivirus-induced lines generating 25.2 ± 7.5 OEP and 22.2 ± 11.3 ONP colonies/cm^2^, while the mRNA-reprogrammed lines produced 15.3 ± 7.8 OEP and 24.4 ± 4.5 ONP colonies/cm^2^ (*n* = 2, *p* > 0.05; Figure 1(d)). The individual yields of each line are presented in supplementary [Supplementary-material supplementary-material-1]. Visual identification of these morphologically distinct subpopulations within cultures is important as manual purification of ONPs and OEPs can be used to select them prior to the second stages of neuronal and hair cell differentiation, respectively.

### 3.2. iPSC-Derived Otic Neuroprogenitors Can Be Induced towards a Neuronal Fate

**Figure 2:**
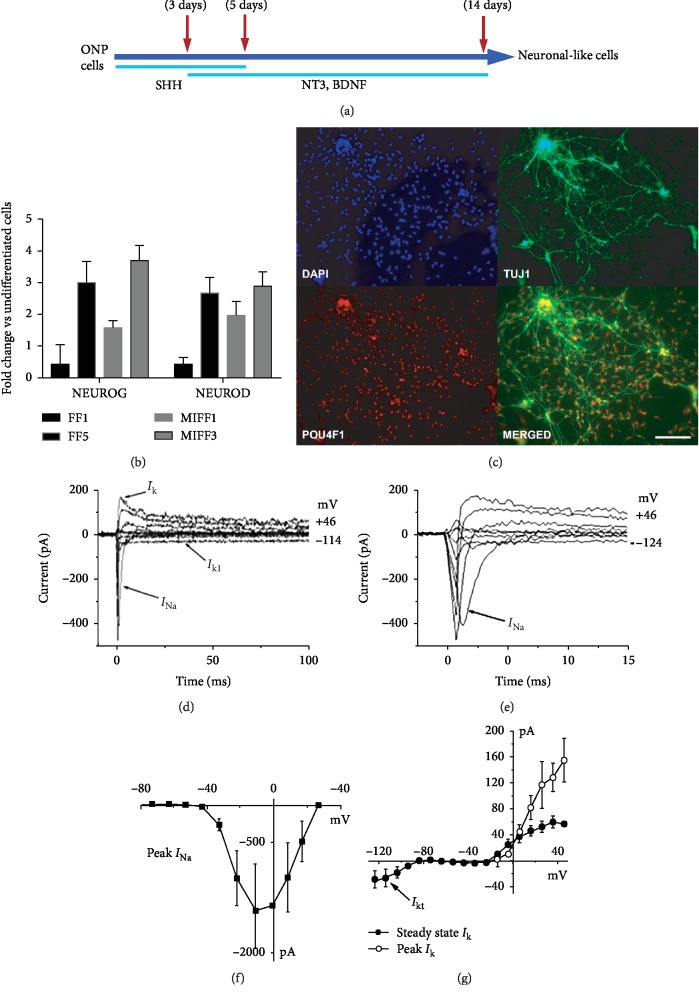
(a) Diagram showing the protocol for the generation of spiral ganglion-like neurons. (b) qPCR showing relative expression levels of *NEUROG* and *NEUROD.* Data shown as fold change against undifferentiated cells from the same initial population. (c) ONPs differentiating into neurons express markers such as TUJ1 and POU4F1, scale bar is 200 *μ*m. Immunofluorescence images shown are from cell line FF5. (d) Electrophysiological properties of differentiating neuronal-like cells. Depolarizing and hyperpolarizing voltage steps in 10 mV nominal increments from a holding potential of -84 mV revealed Na^+^ currents (*I*_Na_; (d), expanded in (e)) in 3 out of 3 neuronal-like cells, small outward K^+^ currents (*I*_K_; (d)) in 3 out of 3 cells and a small inward K^+^ current (*I*_K1_) in 1 out of 3 cells (d). The peak *I-V* curve for *I*_Na_ indicates that *I*_Na_ activated at potentials near -40 mV and reached a maximum size of 1328 ± 634 pA near -10 mV (*n* = 3; (f)). The steady state *I-V* curve for *I*_*K*_ was also generated from a holding potential of -84 mV, and the current size at 0 mV and measured at 160 ms was 32 ± 10 pA, *n* = 3 (g). The small inward K^+^ current (*I*_K1_) was evident for membrane potentials negative to -80 mV (g).

Once purified, ONP cells can be passaged and maintained in a progenitor state. ONPs generated using the iPSC lines were subjected to the differentiation regime defined before using human fetal auditory stem cells (hFASCs) [5] and later applied to hESC-derived otic progenitors [6] to obtain sensory neuron-like cells (Figure 2). When cultured under these conditions, iPSC-derived ONPs expressed neuronal markers such as *β*-tubulin III as well as a transcription factor associated with spiral ganglion neuron development, POU4F1 (BRN3A) (Figure 2(c)). Cells extended neurite projections, making cell networks (Figure 2(c)). The levels of expression of the neuro-otic markers *NEUROG* and *NEUROD* were explored with qPCR. Although the levels of induction—relative to the original undifferentiated starting population from each line—showed some variability across the different lines (Figure 2(b)), there were no overall differences between the reprogramming methods. Electrophysiological recordings in voltage clamp mode, from cells generated from an mRNA-reprogrammed line (MIFF1), showed inward Na^+^ currents (*I*_Na_) and small outward K^+^ currents (*I*_k_; 32 pA ± 10 at 0 mV; *n* = 3). Inward K^+^ currents (*I*_K1_) were also observed in these cells (Figures 2(d)–2(g)).

### 3.3. Otic Epithelial Progenitors from iPSCs Can Be Further Differentiated towards a Hair Cell-like Phenotype

**Figure 3:**
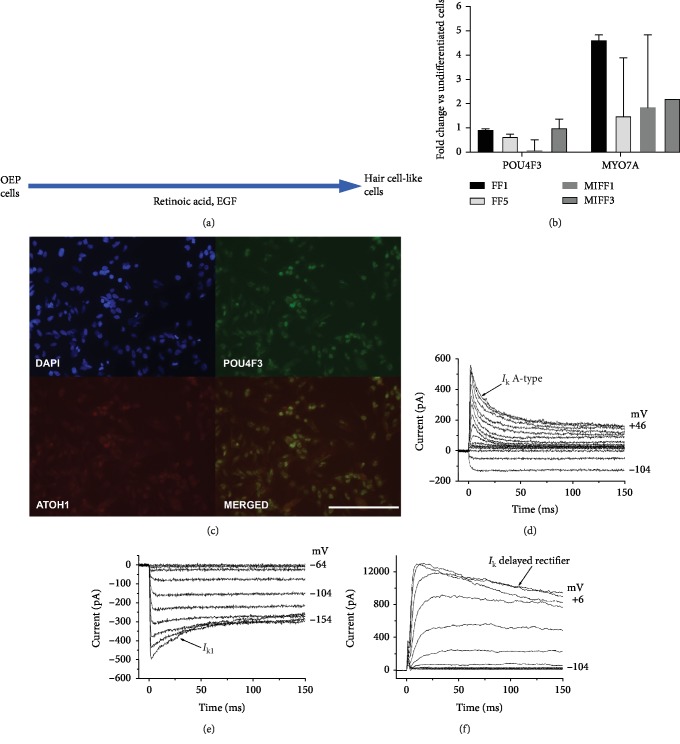
(a) Diagram showing the protocol for the generation of hair cell-like cells. (b) qPCR showing relative expression levels of the hair cell markers *POU4F3* and *MYO7A.* Data shown as fold change against undifferentiated cells from the same initial population. (c) ATOH1 and POU4F3 proteins are coexpressed by some cells. Immunofluorescence images shown are from cell line MIFF1. Scale bar, 200 *μ*m (d). Electrophysiological properties of differentiating hair cell-like cells. To investigate the presence of outward and inward K^+^ currents, cells were recorded using the same voltage protocol described in Figure 2. Outward K^+^ currents were observed in 10 out of 12 hair cell-like cells; these currents were either an inactivating A-type K^+^ current (266 ± 62 pA at 0 mV, *n* = 7 out of 10 cells (d)) or a delayed rectifier current (476 ± 174 pA at 0 mV, *n* = 3 out of 10 cells (f)). Inward K^+^ currents (*I*_K1_: −307 ± 79 pA near -124 mV) were observed in 4 out of 12 hair cell-like cells ((e) holding potential -64 mV, same as the cell in (d)).

OEPs generated through 12 days of FGF3 and FGF10 induction and manually enriched were cultured in the hair-cell-inducing medium reported before [5, 6]. Removal of the FGFs and enhancement with retinoic acid and EGF led to the development of cells arranged in small epithelial colonies, displaying a rearrangement of the actin cytoskeleton, with the formation of a substantial circumferential ring. This morphology has been previously associated with hair cell development in vitro [5]. Relative levels of expression of the hair cell genes *POU4F3* and *ATOH1* were comparable across the different lines showing no differences between reprogramming techniques (Figure 3(b)). Probing these cultures with antibodies against POU4F3 (BRN3C) and ATOH1 indicated coexpression in small subsets (Figure 3(c)). Electrophysiological recordings from cells generated from mRNA reprogramming (MIFF1) revealed outward K^+^ currents in 10 out of 12 cells, which included an inactivating A-type K^+^ current (*n* = 7 out of 10) or a delayed rectifier K^+^ current (*n* = 3 out of 10). Inward K^+^ currents (*I*_K1_) were also observed in 4 out of 12 hair cell-like cells, 2 of which also showed an inactivating A-type K^+^ current (Figures 3(c)–3(e)). These currents resemble those observed in hair cell-like cells derived from hESCs [6], with the inward *I*_K1_ and outward delayed rectifier K^+^ current resembling those recorded in prehearing mouse cochlear hair cells [27]. An inward Na^+^ current was also observed in one of these cells.

## 4. Discussion

An important step towards realising the patient-specific therapeutic potential of iPSCs is the successful generation of the clinically relevant lineages. To our knowledge, this is the first report of the production of otic cells from human iPSCs generated via a mRNA-based nonintegrating method. These findings enhance the prospects of developing the use of iPSC for the treatment of hearing impairment, reducing some of the safety concerns associated with the generation of reprogrammed cell lines. Otic progenitor populations, similar to those obtained from human ESCs, can be generated through FGF-induction via the FGF3 and FGF10 proteins. These cells reproducibly coexpress key markers such as PAX8 and PAX2, SOX2 or FOXG1, with comparable yields between the reprogramming treatments. As further evidence of their otic potential, data has been collected to demonstrate continued differentiation towards both otic neuronal and hair cell fates. The expression of neural and hair cell markers by qPCR did not show a different level of induction between reprogramming methods. Furthermore, cells coexpressing neuronal markers like *β*-tubulin III alongside the transcription factor POU4F1 (BRN3A) were detected in ONP cultures exposed to SHH, NT3, and BDNF, while cells coexpressing ATOH1 and POU4F3 were observed in cultures exposed to retinoic acid and EGF. These findings suggest that, in principle, mRNA-reprogrammed lines are equally capable to produce otic lineages than the conventional, lentivirus-induced ones although some caution must be exercised when extrapolating these results, as only two lines have been studied in each condition.

Cells derived from the nonintegrative line MIFF1 displayed some key physiological responses associated with neurons such as sodium currents, which are required to trigger action potentials. Hair cell differentiation produced immature cell types, similar to those found during development. Some of the electrophysiological properties detected in the iPSC-derived cells were similar to the physiological characteristics recorded *in vitro* from rodent hair cells. For example, the inward *I*_K1_ currents observed in some iPSC-derived hair cell-like cells are transiently expressed during development in both outer and inner cochlear hair cells [27]. Outward delayed rectifier K^+^ currents, which were also observed in some hair cell-like cells, are found throughout development and in mature cochlear hair cells. Nevertheless, the predominant current observed in the hair cell-like cells was the inactivating A-type current, which is found in vestibular hair cells as well as neurons [28, 29], but not in cochlea hair cells.

Induced PSCs are also proving to be effective tools in modelling human disease, helping in our understanding of genetic mutations and drug screening. They have recently started to be applied to the field of otology [24, 25, 30, 31], as platforms to study the correction of naturally occurring genetic mutations that induce deafness. We believe that showing that mRNA, nonintegrative reprogrammed cells can effectively differentiate into otic progenitors, should facilitate their use in these areas, limiting the genetic variability introduced by viral, DNA-based reprogramming. Reprogramming using mRNAs has an additional advantage over viral reprogramming, with iPSCs being attainable in as little as 9 days [32] without then needing to screen for integrations. Although fibroblasts are efficiently reprogrammed by mRNAs, a potential limitation to the widespread application of this technique is the relative difficulty encountered to deliver mRNAs into blood-derived cell types [33], considered at this stage the preferred target cell population to generate iPSCs for therapeutic application. In this regard, the use of Sendai virus [34]—an RNA virus that can deliver genes without integrating—has gained popularity as it can target blood lineages. However, Sendai-mediated reprogramming can leave viral particles contaminating the cells, and being a viral system, a thorough screening for genetic integration or potential mutations should be undertaken before the clinical use of these cells [34]. Since reprogramming by mRNA is a truly “footprint-free” integration system, future work with this technology should be aimed at developing more effective ways to target the blood cell populations. The potential impact that the tissue of origin could have on the ability of mRNA-induced iPSCs to generate otic lineages remains unexplored and will need to be addressed in the future.

## 5. Conclusions

In this study, we present evidence that iPSC lines that have been reprogrammed using nonintegrating mRNAs are capable to differentiate into otic cell types. The ease of generation and lack of genetic integration problems make mRNA-reprogrammed iPSCs highly attractive to the field of regenerative medicine.

## Data Availability

All data used to support the findings of this study are included within the article.

## Abbreviations

iPSCs: Induced pluripotent stem cells
FGF3: Fibroblast growth factor 3
FGF10: Fibroblast growth factor 10
OEP: Otic epithelial progenitor
ONP: Otic neuroprogenitor
hESCs: Human embryonic stem cells
DMEM/F12: Dulbecco's Modified Eagle Medium: Ham's F12
DFNB: DMEM+F12+N2+B27
EGF: Epidermal growth factor
Shh-C24III: Sonic hedgehog
bFGF: Basic fibroblast growth factor
NT3: Neurotrophin-3
BDNF: Brain-derived neurotrophic factor
HEPES: 4-(2-Hydroxyethyl)-1-piperazineethanesulfonic acid
PBS: Phosphate-Buffered Saline
DAPI: 4′,6-Diamidino-2-phenylindole
qPCR: Quantitative real-time polymerase chain reaction
SEM: Standard error of the mean
hFASCs: Human fetal auditory stem cells.

## Supplementary Materials

Supplementary Materials Supplementary Figure 1: pluripotency markers expressed by the lentivirus-induced lines FF1 and FF5. (A) Flow cytometry histograms showing, in red, the percentages of cells expressing SSEA3, SSEA4, TRA1-60, and TRA-1-81. Blue shows the negative, baseline control. (B) Expression of SOX2, OCT4, and NANOG determined by qPCR on samples from the FF1 and FF5 cell lines, as well as from human fibroblasts. Expression levels are presented as 2^-*ΔΔ*Ct^. Supplementary Figure 2: bar charts displaying percentages of double-positive otic progenitors obtained per each individual line, expressing high levels of SOX2/PAX8, FOXG1/PAX8, and PAX2/PAX8. Supplementary Figure 3: bar chart displaying the yields of OEPs and ONPs obtained per individual line.
