# Supplementary material for: Generation of Otic Lineages from Integration-Free Human-Induced Pluripotent Stem Cells Reprogrammed by mRNAs
Source: Stem Cells Int. 2020 Mar 1;2020:3692937. doi: 10.1155/2020/3692937 (PMC7068143; doi:10.1155/2020/3692937)
Supplement: Supplementary Materials — Supplementary Figure 1: pluripotency markers expressed by the lentivirus-induced lines FF1 and FF5. (A) Flow cytometry histograms showing, in red, the percentages of cells expressing SSEA3, SSEA4, TRA1-60, and TRA-1-81. Blue shows the negative, baseline control. (B) Expression of SOX2, OCT4, and NANOG determined by qPCR on samples from the FF1 and FF5 cell lines, as well as from human fibroblasts. Expression levels are presented as 2-ΔΔCt. Supplementary Figure 2: bar charts displaying percentages of double-positive otic progenitors obtained per each individual line, expressing high levels of SOX2/PAX8, FOXG1/PAX8, and PAX2/PAX8. Supplementary Figure 3: bar chart displaying the yields of OEPs and ONPs obtained per individual line. [file 3692937.f1.pdf]

Supplementary figures

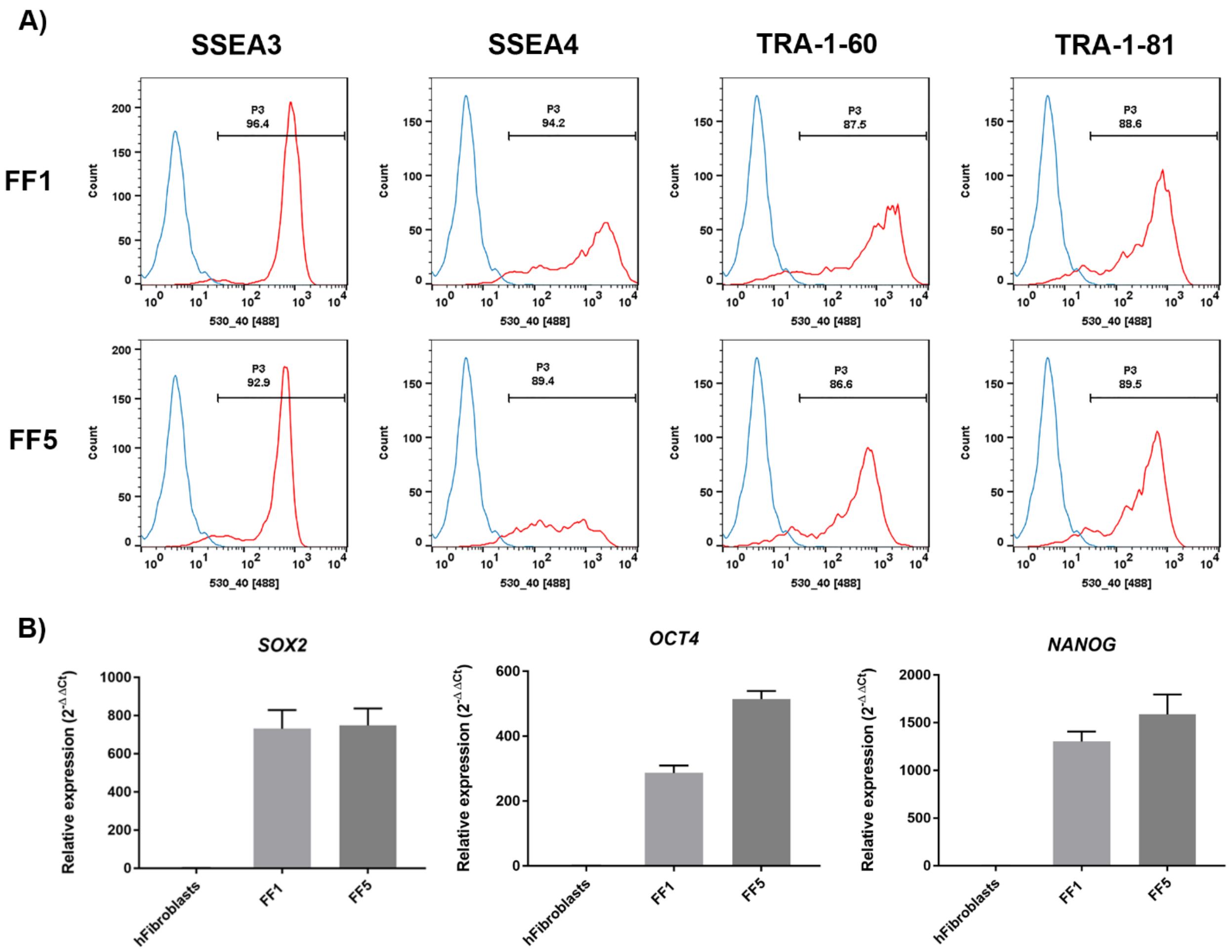

Supplementary Figure 1. Pluripotency markers expressed by the lentivirus-induced lines FF1 and FF5. A) Flow cytometry histograms showing, in red, the percentages of cells expressing SSEA3, SSEA4, TRA-1-60 and TRA-1-81. Blue shows the negative, baseline control. B) Expression of *SOX2, OCT4* and *NANOG* determined by qPCR on samples from the FF1 and FF5 cell lines, as well as from human fibroblasts. Expression levels are presented as $2^{-\Delta\Delta Ct}$.

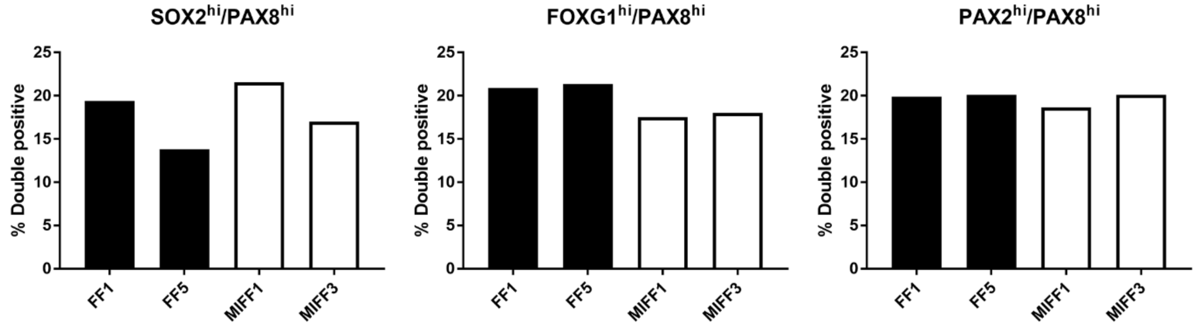

Supplementary Figure 2. Bar charts displaying percentages of double positive otic progenitors obtained per each individual line, expressing high levels of SOX2/PAX8; FOXG1/PAX8 and PAX2/PAX8.

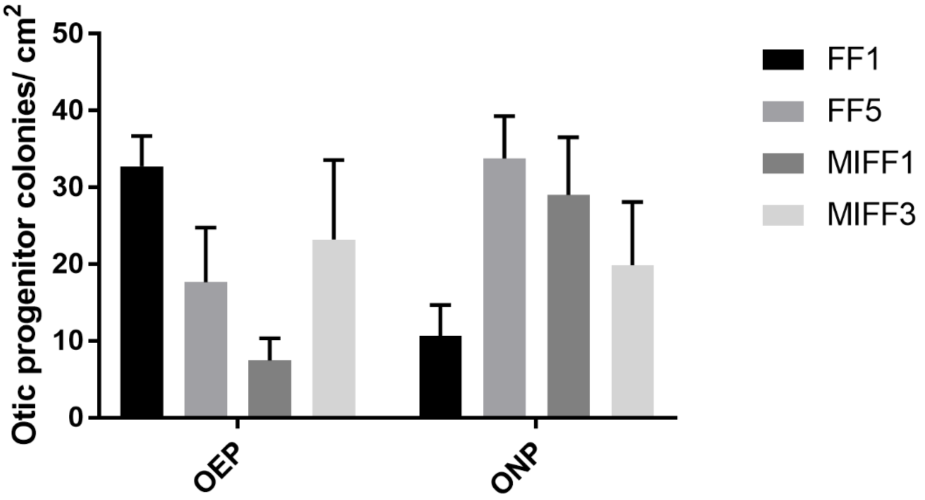

Supplementary Figure 3. Bar chart displaying the yields of OEPs and ONPs obtained per individual line.
